# Development and Optimization of a Fluorescent Differential Display PCR System for Analyzing the Stress Response in *Lactobacillus sakei* Strains

**DOI:** 10.3390/nu1020210

**Published:** 2009-11-30

**Authors:** Maria Grazia Bonomo, Maria Anna Sico, Simona Grieco, Giovanni Salzano

**Affiliations:** Dipartimento di Biologia, Difesa e Biotecnologie Agro-Forestali, Università degli Studi della Basilicata, Viale dell’Ateneo Lucano, 10, 85100 Potenza, Italy; Email: mariaanna.sico@unibas.it (M.A.S.); giovanni.salzano@unibas.it (G.S.); simona@viavai.com (S.G.).

**Keywords:** Fluorescent Differential Display PCR, *Lactobacillus sakei*, stress response

## Abstract

*Lactobacillus sakei* is widely used as starter in the production process of Italian fermented sausages and its growth and survival are affected by various factors. We studied the differential expression of genome in response to different stresses by the fluorescent differential display (FDD) technique. This study resulted in the development and optimization of an innovative technique, with a high level of reproducibility and quality, which allows the identification of gene expression changes associated with different microbial behaviours under different growth conditions.

## 1. Introduction

Lactic acid bacteria are widely used in meat fermentation and an efficient control of these microbiological processes requires increased knowledge of bacterial behaviour under stress conditions. *Lactobacillus (Lb.)* *sakei* is recognized as one of the most important components of starter cultures used for production of Italian fermented sausages and its growth and survival are affected by various factors such as temperature, pH and salt concentration [1]. Recently, the genome of the sausage isolate *L. sakei* 23K was studied, analyzed [2] and then its sequence was published [3] and further investigated [4], providing fundamental information on the genetic makeup of this organism. 

Several techniques, such as differential display (DD-PCR) [5] and RNA arbitrarily primed polymerase chain reaction (RAP-PCR) [6], have been developed and have now become routine to interpret gene expression patterns in different biological systems. The DD-PCR method was originally developed for use in the study of eukaryotic gene expression, and consists of cDNA production from mRNA that has been reversely transcribed with different primers anchored to the polyadenylated tail, followed by amplification of cDNAs with arbitrary primers, with incorporation of a radioactive label and electrophoresis in polyacrylamide sequencing gels [5,6,7]. RAP-PCR utilizes an arbitrary primer at a low annealing temperature for cDNA synthesis reactions, so it was used for amplification of RNAs that are not polyadenylated, such as bacterial RNA [6]. This technology has been applied to only a few prokaryotic systems [8,9,10,11,12,13]. 

Fluorescent differential display (FDD) represents the next logical progression which use labeled primers or direct incorporation of labeled dNTPs and have been widely used and have replaced radioactive detection in many procedures [14,15]. FDD, optimized using fluorochrome labeled anchor primers and higher dNTP concentrations in PCR, was shown to be essentially identical in both sensitivity and reproducibility to that of conventional DD. Improvements such as elimination of radioactivity, digital data acquisition and increased assay speed were goals that were successfully achieved with the establishment of the FDD platform, representing a marked improvement over conventional DD [16]. The FDD technique was used in some studies [17,18,19,20] and this methodology allows the examination of changes in gene expression in response to different situations without the need of any prior knowledge of genomic information or selecting candidate genes that may be involved in the stress mechanisms. In this way, novel genes may be identified, as already occurred in several previous studies [17,18,21,22]. 

We have tried to optimize and standardize an assay system based on the FDD method to apply it to prokaryotic systems in order to identify gene expression changes associated with differential microbial behaviours under different growth conditions with a rapid, simple test and with a high level of reproducibility and quality. Thus, the aim of this study was to develop and optimize the FDD method for analyzing the different expression fingerprints of *Lb. sakei* in order to verify the applicability of the selected potential starters and to obtain a better stress response definition in this species. We have developed an innovative FDD-PCR protocol that allows us to identify differently expressed transcripts and to compare the difference of cDNA fingerprints when different treatments and time periods are involved.

## 2. Results and Discussion

Fluorescent differential display profiles were produced using an assay system based on the FDD method, by employing a single fluorescently labeled random primer for detecting and isolating differentially expressed fragments under different stress situations. 

**Figure 1:**
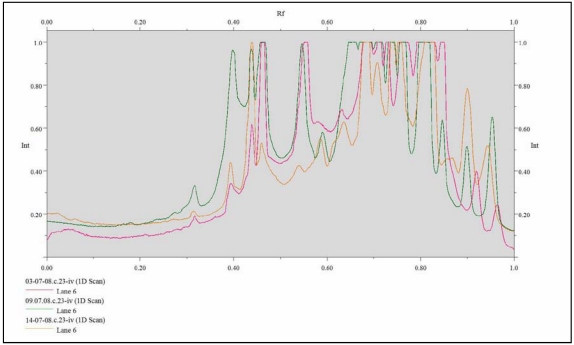
Compared lane analysis of the three profiles for a representative strain (DBPZ0329) response to a stress condition.

It has been necessary to first perform a series of laboratory tests in order to optimize the work protocol and to standardize the method for our research, since the differential display amplification of RNA from bacteria cultured independently under identical conditions produced widely different product patterns. Some modifications, detailed in the following section, were made to the standard protocol to obtain a reproducible and standardized method to identify differences in genome expression. First of all, three concentrations of RNA were evaluated to overcome the problem of concentration-dependent amplification. Although broadly similar from lane to lane, there were RNA-dependent differences in the number of PCR products detected. A plateau in the number of well resolved and easily detected products was observed at about 2 μg of RNA, so this RNA concentration was selected for use in the cDNA synthesis reaction (data not shown). Moreover, as FDD-PCR depends on the random priming of a universal random primer at low annealing temperature, the effect of different annealing temperatures in the second-strand synthesis reaction was investigated. The second-strand annealing temperature was increased from 30 to 42 °C and, with interest in maximizing both the number and the resolution of PCR products, in the end 36 °C was chosen as the second-strand annealing temperature in subsequent reactions. Equilibration to a specific annealing temperature prior to the addition of *Taq* polymerase is essential for eliminating the variability inherent to random priming; thus, each sample was randomly primed essentially to completeness prior to the initiation of polymerization, and polymerization was initiated under identical conditions (data not shown). The reproducibility of the FDD technique described in the present work was assessed performing the PCR reactions for each condition tested in triplicate and comparing the obtained fingerprints by compare lane analysis. Figure 1 shows a comparative lane analysis of the three profiles for a representative strain response to one of the stress conditions. It was observed that three independent but identical amplifications of each RNA preparation yielded identical product patterns; the complete peak overlap represents the presence of the same bands in all fingerprints produced, demonstrating the high reproducibility of the method used. We carried out this analysis for each strain with an high reproducibility of the method by generating the same amplification patterns from the same cDNA samples. The analysis of the tracing peaks was performed by Diversity Database^TM^ software and it gave 100% similarity among them, validating the experimental procedure. 

Thus, this work established and optimized a reproducible method that enables the identification of different expression changes of *Lb. sakei* in stress conditions, with a reduction in false positives, as already observed in other studies [17,18,19,20], and the assessment that the use of 6-FAM-M13 primer resulted in satisfactory band intensity for PCR products, allowing us to also visualize smaller bands as already noted by other authors [9,11,12,17,18,20].

Distinct bands were obtained in response to different stresses, such as low pH values, elevated temperatures and high osmolarity, and the identification of their sequences is currently under way. The resulting fingerprints showed the presence and the absence of specific products between each stressed sample and the control. \Table 1 shows the fingerprints of each *Lb. sakei* strain; we can see the band sizes of the specific amplification products present in the control and in the different stressed samples. These products were compared with bands already reported in the literature for stress conditions [4,23,24,25] and we observed the presence of some bands only in the stressed samples with a molecular weight that can be referred to specific genes under stress situations. 

**Table 1:** Fingerprints of *Lb. sakei* strains under stress conditions. The values indicate a molecular weight (expressed in base pairs) of the bands find in the control and in each stressed sample.

		DBPZ0098				
Control	50°C	55°C	60°C	pH 2.5	pH 3	NaCl 9%
1673.44		1673.44			1673.44	1673.44
	**1328.16**		1328.16	1328.16		
		**1018.72**				
		**931.93**	931.93	931.93		
882.75	882.75	882.75	882.75	882.75	882.75	
864.42	864.42					864.42
834.89	834.89	834.89	834.89	834.89		834.89
784.89	784.89	784.89	784.89	784.89	784.89	
749.74		749.74	749.74	749.74		749.74
693.97	693.97	693.97	693.97	693.97	693.97	693.97
663.45		663.45	663.45	663.45	663.45	
	**632.92**	632.92				
587.53	587.53	587.53	587.53	587.53		587.53
	**550.05**					
530.28	530.28	530.28	530.28	530.28	530.28	530.28
504.74	504.74	504.74	504.74	504.74	504.74	504.74
	**486.83**	486.83				
469.53	469.53	469.53	469.53	469.53	469.53	469.53
452.86	452.86	452.86	452.86	452.86	452.86	452.86
417.27	417.27	417.27	417.27	417.27	417.27	417.27
**DBPZ0338**						
**Control**	**50°C**	**55°C**	**60°C**	**pH 2.5**	**pH 3**	**NaCl 9%**
						
2560.91	2560.91	2560.91	2560.91	2560.91		2560.91
		**1750.63**				
		**1450.49**				
980.06			980.06		980.06	
	**970.73**		970.73			
						**851.94**
773.31	773.31	773.31			773.31	
	**738.01**					
704.32	704.32		704.32	704.32	704.32	704.32
674.71	674.71		674.71		674.71	674.71
600.51	600.51	600.51	600.51	600.51	600.51	600.51
571.08	571.08	571.08	571.08	571.08	571.08	571.08
532.01	532.01	532.01	532.01	532.01	532.01	532.01
513.04	513.04	513.04	513.04	513.04	513.04	513.04
336.57	336.57	336.57	336.57	336.57	336.57	336.57
**DBPZ0416**						
**Control**	**50°C**	**55°C**	**60°C**	**pH 2.5**	**pH 3**	**NaCl 9%**
						
	**6032.41**					
5370.14		5370.14	5370.14		5370.14	5370.14
					**3653.56**	
3292.21		3292.21	3292.21		3292.21	3292.21
2721.33	2721.33		2721.33	2721.33		2721.33
2473.01	2473.01	2473.01	2473.01		2473.01	2473.01
2383.11			2383.11		2383.11	2383.11
	**2276.34**	2276.34		2276.34		
2184.14			2184.14	2184.14	2184.14	2184.14
2022.33	2022.33	2022.33	2022.33		2022.33	2022.33
1875.22	1875.22	1875.22	1875.22	1875.22	1875.22	1875.22
1771.13	1771.13	1771.13	1771.13	1771.13	1771.13	1771.13
1720.02	1720.02	1720.02	1720.02	1720.02	1720.02	1720.02
1642.11	1642.11	1642.11	1642.11	1642.11	1642.11	1642.11
1544.23	1544.23			1544.23	1544.23	1544.23
1454.23	1454.23	1454.23	1454.23	1454.23	1454.23	1454.23
1323.41	1323.41	1323.41	1323.41		1323.41	1323.41
1151.11		1151.11				1151.11
	**1074.03**					
880.12		880.12	880.12	880.12	880.12	880.12
863.45	863.45	863.45	863.45		863.45	863.45
740.53						
	**643.75**	643.75	643.75	643.75		
600.21	600.21	600.21	600.21	600.21	600.21	600.21
	**576.43**					
512.01	512.01	512.01	512.01	512.01	512.01	512.01
486.52	486.52	486.52	486.52	486.52	486.52	486.52
478.59		478.59	478.59			478.59
441.67			441.67	441.67		
412.34	412.34		412.34	412.34		
				**387.54**		
365.55	365.55	365.55	365.55	365.55		365.55
		354.22	354.22		354.22	
	**304.53**			304.53		
	**291.23**			291.23		291.23
**DBPZ0062**						
**Control**	**50°C**	**55°C**	**60°C**	**pH 2.5**	**pH 3**	**NaCl 9%**
						
		**5461.79**				
3862.88	3862.88		3862.88			3862.88
		**3333.04**				
				**2441.51**		2441.51
1685.79						1685.79
1503.95	1503.95	1503.95		1503.95		
1166.63	1166.63	1166.63	1166.63	1166.63		1166.63
		**1039.42**		1039.42		
964.55						
901.32	901.32	901.32	901.32	901.32	901.32	901.32
870.74	870.74					870.74
840.71					840.71	
796.05	796.05	796.05	796.05	796.05		
705.81						
687.53	687.53	687.53	687.53	687.53	687.53	687.53
649.93	649.93	649.93	649.93		649.93	649.93
		**636.22**		636.22		
615.23						
607.29	607.29	607.29	607.29	607.29	607.29	607.29
584.36	584.36	584.36	584.36	584.36	584.36	584.36
543.07	543.07	543.07	543.07	543.07	543.07	543.07
511.96	511.96	511.96	511.96	511.96	511.96	
			**497.97**		497.97	
	**472.33**			472.33	472.33	472.33
					451.2	
433.57	433.57	433.57	433.57	433.57	433.57	433.57
**DBPZ0329**						
**Control**	**50°C**	**55°C**	**60°C**	**pH 2.5**	**pH 3**	**NaCl 9%**
						
			**1722.17**	1722.17		
				1468.41		
			**1422.94**			
			**1308.32**			
1022.48	1022.48	1022.48	1022.48	1022.48	1022.48	1022.48
			**965.71**			965.71
		943.36	943.36	943.36	943.36	943.36
873.34		873.34	873.34	873.34	873.34	873.34
			840.95			
805.84					805.84	
				**779.32**		779.32
759.71		759.71	759.71			
746.03		746.03			746.03	
			708.68	708.68	708.68	
		702.39		702.39	702.39	702.39
697.99			697.99	697.99	697.99	697.99
		**673.84**				
		663.54	663.54	663.54	663.54	663.54
659.26		659.26	659.26		659.26	659.26
624.48	624.48	624.48	624.48	624.48	624.48	624.48
		**597.42**	597.42			597.42
		**570.58**			570.58	570.58
548.1	548.1	548.1	548.1	548.1	548.1	548.1
				**546.18**		546.18
		527.78	527.78	527.78	527.78	
522.14		522.14	522.14	522.14		522.14
501.79	501.79	501.79	501.79	501.79	501.79	501.79
494.21	494.21			494.21		
479.58	479.58	479.58	479.58	479.58	479.58	479.58
		**470.79**				
459.58	459.58	459.58	459.58	459.58	459.58	459.58
		**448.37**				
414.21	414.21		414.21	414.21	414.21	414.21
389.06	389.06	389.06	389.06	389.06	389.06	389.06

**Figure 2:**
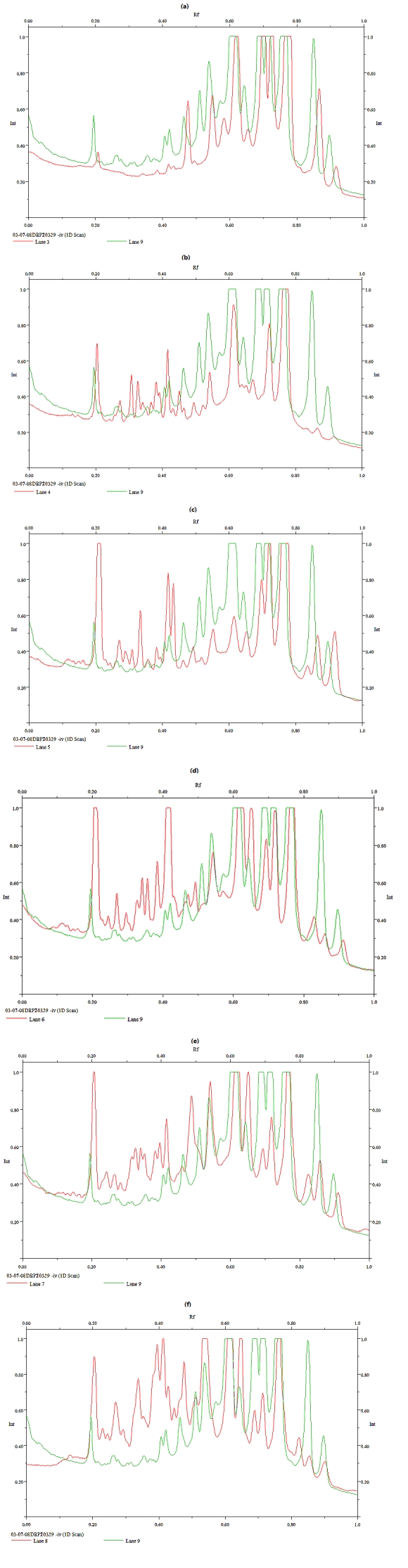
Analysis of the fingerprints obtained by comparing each lane of the stressed sample with that of the control. (a) Stressed sample at 50 °C and the control. (b) Stressed sample at 55 °C and the control. (c) Stressed sample at 60 °C and the control. (d) Stressed sample at pH 2.5 and the control. (e) Stressed sample at pH 3 and the control. (f) Stressed sample at 9% NaCl and the control.

As a random analysis on mRNA transcript was performed, it might be possible that some fragments obtained are part of genes known or not yet known, so we decided to compare accurately each lane of the stressed sample with that of the control (Figure 2) in order to analyze and choose the more interesting bands. 

As shown in this Figure, the analysis demonstrated the expression of specific products by comparison of tracings related to the different samples lanes in particular stress conditions. The compare lane analysis proved the presence of some bands and the absence of others, so it evidenced that, in order to survive and adjust to the stresses encountered, *Lb. sakei* strains respond to stimuli through the differential expression of transcripts. Bands of interest (labelled in bold in Table 1) were selected, recovered and re-amplified by standard PCR with the same universal primer used in FDD-PCR. After re-amplification, the products were gel purified and sent for sequencing and their identification is currently underway. The results of this analysis confirmed the effectiveness of the system at identifying differentially expressed transcripts on FDD fingerprints correlated with microbial tolerance to stress situations. Sequence and expression studies are currently under way and then an additional search is required to gather information essential to determine the functional role of these genes.

## 3. Experimental Section

### 3.1. Bacterial Strains and Culture Conditions

Five strains of *Lb. sakei* (DBPZ0062, DBPZ0098, DBPZ0338, DBPZ0329 and DBPZ0416) from the culture collection of the Dipartimento di Biologia, Difesa e Biotecnologie Agro-Forestali, Università degli Studi della Basilicata, Potenza, Italy, used in this study were isolated from traditional fermented sausages of the Basilicata region and chosen as potential autochthonous starters in a previous study [26]. All strains were maintained as freeze-dried stocks in reconstituted (11% w/v) skim milk, containing 0.1% w/v ascorbic acid (Riedel-de Haën, Sigma-Aldrich, Milan, Italy) and routinely cultivated in MRS broth at 30 °C for 16 h, before the evaluation of stress response.

### 3.2. Stress Treatments

Late-exponential phase cells grown overnight in MRS broth, pH 6.2, were harvested by centrifugation (12,000 rpm, 5 min) and washed twice in sterile saline solution (0.85 % w/v NaCl). The cells were re-suspended in 2 mL of different media to a final OD_600_ = 1.0 to achieve the following stress conditions: (a) MRS broth, pH 2.5 and 3.0 (adjusted with HCl, acid stress); (b) MRS broth with 9% w/v NaCl (osmotic stress); (c) MRS broth, pH 6.2, at different temperatures (heat stress). Cell suspensions were incubated for 30 min at 30 °C for acid and osmotic stresses and at 50, 55 and 60 °C for heat stress. Bacterial cells incubated at 30 °C for 30 min in 2 mL of MRS broth, pH 6.2, were used as controls.

### 3.3. Fluorescent Differential Display (FDD)

RNA was extracted from 2 mL of *Lb. sakei* culture using RNA isolation kit supplied by Gentra System, Inc. (Minneapolis, MN, USA) according to the manufacturer’s instructions. Purified RNAs were suspended in 200 μL of DEPC 0.1% diethylpyrocarbonate-treated water and stored at −20 °C. RNAs concentrations were calculated by measuring absorbance at 260 nm using the NanoDrop® ND-1000 spectrophotometer (Nanodrop Technologies Inc.). Prior to reverse transcription (RT), RNA (2 μg of total RNA) was treated with 2U/ μL of RNase-free DNase (Ambion, Applied Biosystems, Austin, TX, USA), as described by the manufacturer, to achieve complete DNA removal. Then, the cDNA was synthesized using the ProSTAR^TM^ First-Strand RT-PCR kit (stratagene, La Jolla, CA, USA) as recommended. RT reactions were performed in a final volume of 150 μL that contained master mix, random primers and 10 μL of RNA by subsequent incubation at 80 °C for 3 min, at 42 °C for 2 h and at 16 °C for 2 h. After diluition, 2 μL of cDNA were used for PCR analyses.

The FDD technique was carried out by using a single fluorescently labeled universal primer, the random 6-carboxyfluorescein labeled 5’-anchored M13 primer (5’-6-carboxyfluorescein (FAM)-GAGGGTGGCGGTTCT-3’). The PCR mixture (25 μL) consisted of 2 μL of diluted cDNA, 2.5 μl of 1X PCR buffer (EuroClone, Pero, Milano, Italy), 3 mM of MgCl_2_ (EuroClone), 0.4 mM of each dNTP (EuroClone), 0.6 μM of the primer (Invitrogen Ltd, Paisley,UK), 2.5 U of *Taq* polymerase (EuroClone). PCR amplification was carried out in a Genius Techne Progene thermal cycler (Cambridge, UK) using the following program: initial denaturation at 94 °C for 1 min; 40 cycles of 94 °C for 1 min, annealing at 36 °C for 1 min and extension at 72 °C for 2 min; followed by a final extension at 72 °C for 7 min. The PCR products were separated by electrophoresis on 2% (w/v) agarose gels (EuroClone) in 1X TBE at 100 V for 4 h. Gels were stained in 1X TBE buffer containing 0.5 μg/mL ethidium bromide (Serva Electrophoresis GmbH, Heidelberg, Germany) for 30 min. A 1 kb DNA ladder (EuroClone) was used as molecular weight and normalization gel standard. 

The banding patterns were visualized by UV transillumination and captured with GelDoc 2,000 Apparatus (BioRad). Gel images were digitized in Diversity Database^TM^ software (Bio-Rad Laboratories Ltd., Watford, Herts, UK) and processed for analysis and selection of the fragments. 

PCR reactions were done in triplicate and the comparison of obtained fingerprints was carried out by using the compare lane analysis feature of the Diversity Database^TM^ software to demonstrate the reproducibility of the method used and also to analyze and choose more accurately patterns obtained under different conditions tested.

## 4. Conclusions

In this study, the development and the optimization of a technique able to identify gene expression changes, associated with differential microbial behaviours under different growth conditions, was achieved. In this work we tested five *Lb. sakei* strains chosen as potential autochthonous starter cultures on the basis of genetic diversity and important technological properties [26] and also more competitive, well adapted to the particular product and to the specific production technology and with high metabolic capacities. The aim was to examine the effective capacity of these strains to withstand and tolerate different stress conditions because the formulation and preservation of starter cultures may impose environmental stresses on the bacterial cells which influence the metabolic activities and decrease the performance of the starters in industry biotechnology. Therefore, the development of rapid and efficient method for studying the effect of stress on bacterial cells is important to verify the applicability of starter cultures subjected to different stresses.

The availability of a reliable method for the differentiation of *Lb. sakei* strains is indispensable for a better definition of genotypes developed in different regions of fermented sausages production, with an advance in the evaluation of the genomic differences among strains in order to examine the evolution of this species. Thus, the application of an innovative and promising FDD method, with an high level of reproducibility and quality and the refinements detailed in the present work, allows one to carry out a specific FDD-PCR protocol to serve as a general, highly reproducible and standardized technique for studying and probing the knowledge of the relation between differential genome expression and tolerance to different stresses.

The FDD-PCR isn’t a quantitative technique, however, so if and when deemed necessary, some results will be also analyzed by qPCR to achieve further information about the expression level of a particular gene in different conditions, but without invalidating the simplicity of execution and the innovation of the technique applied. The analysis of the transcripts sequences and the investigation of their products and of their role in the stress response and their involvement in physiological changes of bacterial behaviour were the next step of our research.
